# An acquired BMF with FANCL gene heterozygous mutation: Case report

**DOI:** 10.1097/MD.0000000000034036

**Published:** 2023-06-16

**Authors:** Nan Zhang, Xiao Wang, Xiao-Juan Miao, Xu-Pai Zhang, Xin-Yu Xia, Li Li, Hao-Ping Sun

**Affiliations:** a Department of Hematology, People’s Liberation Army The General Hospital of Western Theater Command, Chengdu, China.

**Keywords:** bone marrow failure (acquired BMF), case report, FA complementation gene (FANC gene), FA complementation group L (FANCL)

## Abstract

Bone marrow failure (BMF) includes inherited and acquired BMFs. Acquired BMF can be secondary to various factors, such as autoimmune dysfunction, benzene, drugs, radiation, viral infection and so on. Fanconi anemia (FA) complementation group L (FANCL) is an E3 ubiquitin ligase that participates in the repair of DNA damage. Homozygous or compound heterozygous mutations of FANCL can lead to the onset of FA, which is one of the most common inherited BMFs.

**Patient concerns and Diagnoses:**

Here, we report a case of acquired BMF. This patient had a history of benzene exposure for half a year before the onset of the disease, and presented with progressive pancytopenia, especially the reduction of erythrocytes and megakaryocyte, without malformation. Interestingly, this patient and his brother/father had a heterozygous (non-homozygous/compound heterozygous) mutation (Exon9, c.745C > T, p.H249Y) in the FANCL gene.

**Interventions and Outcomes:**

The patient successfully underwent unrelated and fully compatible umbilical cord blood hematopoietic stem cell transplantation.

**Lessons subsections:**

We report for the first time an acquired BMF case with FANCL gene heterozygous mutation, and the mutation site (Exon9, c.745C > T, p.H249Y) has never been reported. This case suggests that heterozygous mutations in FANCL gene may be associated with increased susceptibility to acquired BMF. Based on current reports and this case, we speculate that heterozygous mutations in the FA complementation gene may exist in a certain proportion of tumor and acquired BMF patients, but have not been detected. We recommend routine screening for FA complementation gene mutations in tumor and acquired BMF patients in clinical practice. If positive results are found, further screening can be conducted on their families.

## 1. Introduction

Acquired bone marrow failure (BMF) can be caused by a variety of factors, such as autoimmune dysfunction, benzene, drugs, radiation, viral infection and so on.^[1,2]^ This case of acquired BMF may be secondary to exposure to benzene. However, interestingly, the next-generation sequencing revealed that the patient had a heterozygous mutation (Exon9, c.745C > T, p.H249Y) in FA complementation group L (FANCL) gene.

FANCL is a key gene involved in the FA DNA repair pathway, and its homozygous or compound heterozygous mutation can lead to the onset of Fanconi anemia (FA), which is one of the most common inherited BMFs.^[3,4]^ FANCL is just one of the FA complementation (FANC) genes, which include a total of 22 genes, namely FANCA, FANCB, FANCC, FANCD1, FANCD2, FANCE, FANCF, FANCG, FANCI, FANCJ, FANCL, FANCM, FANCN, FANCO, FANCP, FANCQ, FANCR, FANCS, FANCT, FANCU, FANCV, and FANCW.^[5–7]^ Researches have reported that heterozygous mutations in the FANC genes may lead to an increased susceptibility to tumors and acquired BMF.^[8–11]^ However, there is currently no literature reporting the relationship between FANCL heterozygous mutation and the onset of acquired BMF. We have reported for the first time a case of acquired BMF with FANCL heterozygous mutation, and the site of this mutation has never been reported.

## 2. Methods

The study was approved by the Ethics Committee of People’s Liberation Army The General Hospital of Western Theater Command.

The authors confirm that “written informed consent” was obtained from the patient’s legal guardian.

## 3. Patient information

The patient, male, fainted after physical exercise in April 2021 (16 years old), and routine blood test showed that white blood cell count (WBC) was 4.16*10^9/L, hemoglobin concentration (HGB) was 89 g/L, platelet count (PLT) was 27*10^9/L. The morphology of bone marrow (BM) showed that the proliferation of nucleated cells was active, and numbers of megakaryocytes and platelets were decreased. No abnormality is found in chromosome karyotype. The patient’s condition did not improve after oral treatment with Leucogen/caffeic acid. Thereafter, the PLT of the patient remained at 25 ± 5*10^9/L for a long time.

The patient went to the outpatient department of our hospital on October 2022. The result of routine blood test showed that WBC was 3.4*10^9/L, HGB was 85 g/L, PLT was 16*10^9/L, average red blood cell volume was 114.5 fL, and average red blood cell hemoglobin content was 37.8 pg, reticulocyte count was 0.034*10^12/L. The second morphology of BM showed that the proliferation of nucleated cells was active, and there were few megacaryocytes and platelets. BM biopsy showed low proliferation of BM tissue. We gave the treatment of glucocorticoid and cyclosporine successively. During the above treatment, the patient developed upper respiratory tract infection with fever in November 2022. The patient underwent routine blood test again. The result showed that WBC (2.16*10^9/L), HGB (78g /L), and PLT (5*10^9/L) further decreased.

## 4. Clinical findings

**Figure 1.**
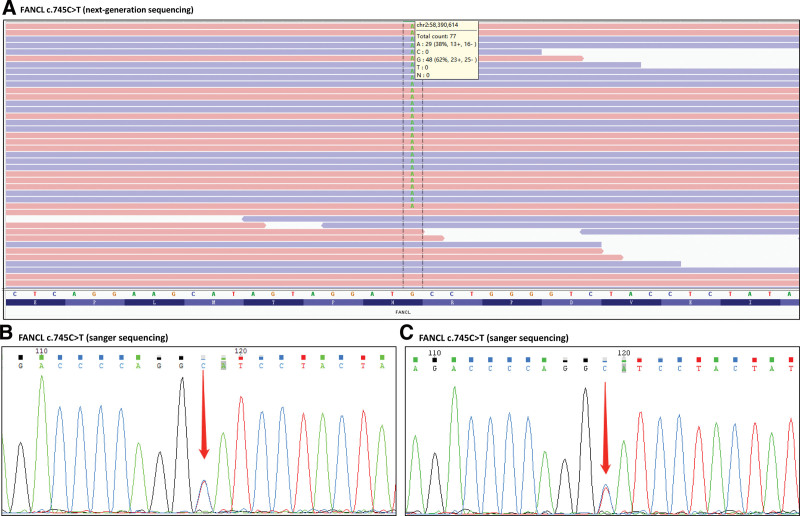
DNA mutation site of FANCL gene of the patient and his brother/father. (A) The NGS of the patient. The sites marked with dotted lines are the mutation sites. (B and C) The sanger sequencing of patient’s brother (B) and father (C). The sites marked with red arrows are the mutation sites. FANCL = FA complementation group L, NGS = next-generation sequencing.

The patient presented with an anemic appearance, without ecchymosis or malformation, and no other special positive signs. The third morphology of BM showed that the proliferative of nucleated cells was low, toxic particles and vacuoles can be seen in the cytoplasm of some neutrophils. BM biopsy showed low proliferation of BM tissues. The patient’s liver function (including bilirubin and transaminase), chromosome karyotype, paroxysmal nocturnal hemoglobinuria screening, virus screening (including COVID-19, cytomegalovirus, Epstein-Barr virus, herpes simplex virus, human T-cell lymphotropic virus, hepatitis B virus, hepatitis C virus, and parvovirus B19), autoimmune antibody, tumor markers, myelodysplastic syndrome-fluorescence in situ hybridization, acute myeloid leukemia (AML) fusion gene and gene mutation, and the mitomycin assay were all negative. However, the next-generation sequencing of inherited Bone marrow failure related genes showed FANCL heterozygous mutation (Exon9, c.745C > T, p.H249Y) (Fig. 1A). His brother and father had the same FANCL heterozygous mutation (Fig. 1B and C). His mother has passed away and her genetic condition is unavailable.

## 5. Timeline

**Figure 2.**
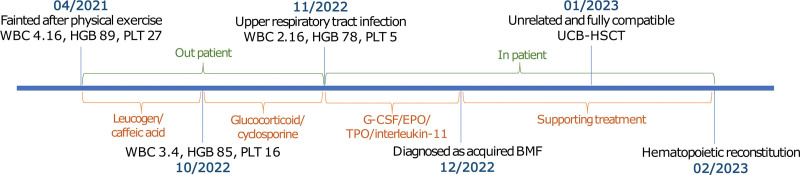
Timeline of this case. BMF = bone marrow failure, HGB = hemoglobin concentration, PLT = platelet count, WBC = white blood cell count.

The timeline is shown in Figure 2.

## 6. Diagnostic assessment

The patient presented with progressive pancytopenia and underwent a series of tests, all of which were negative. So, we considered diagnosing the patient as BMF. The patient had heterozygous mutation in the FANCL gene, rather than homozygous or compound heterozygous mutation, which can lead to the onset of FA. Therefore, the patient is not considered to be diagnosed with FA. With a 6-month history of benzene exposure before the onset of the disease, the patient is considered to be diagnosed as acquired BMF with FANCL heterozygous mutation.

## 7. Therapeutic intervention, follow-up and outcomes

After admitting to inpatient department of our hospital, the patient received symptomatic treatment such as platelet transfusion, anti-infection and granulocyte colony-stimulating factor/erythropoietin/thrombopoietin/interleukin-11 subcutaneous injection. The patient’s Leucocyte count can be elevated under granulocyte colony-stimulating factor treatment. Then, the patient successfully underwent unrelated and fully compatible umbilical cord blood hematopoietic stem cell transplantation (UCB-HSCT). The patient has completed the UCB-HSCT for over 2 months and is currently in good condition.

## 8. Discussion

We conducted a comprehensive examination after discovering that the patient had decreased whole blood cells. After excluding other diseases and considering the patient’s history of benzene exposure, we diagnosed the patient with acquired BMF. We further conducted next-generation sequencing and found that the patient had a heterozygous mutation in the FANCL gene. We also screened for FANCL gene mutation in his family and obtained positive results.

**Figure 3.**
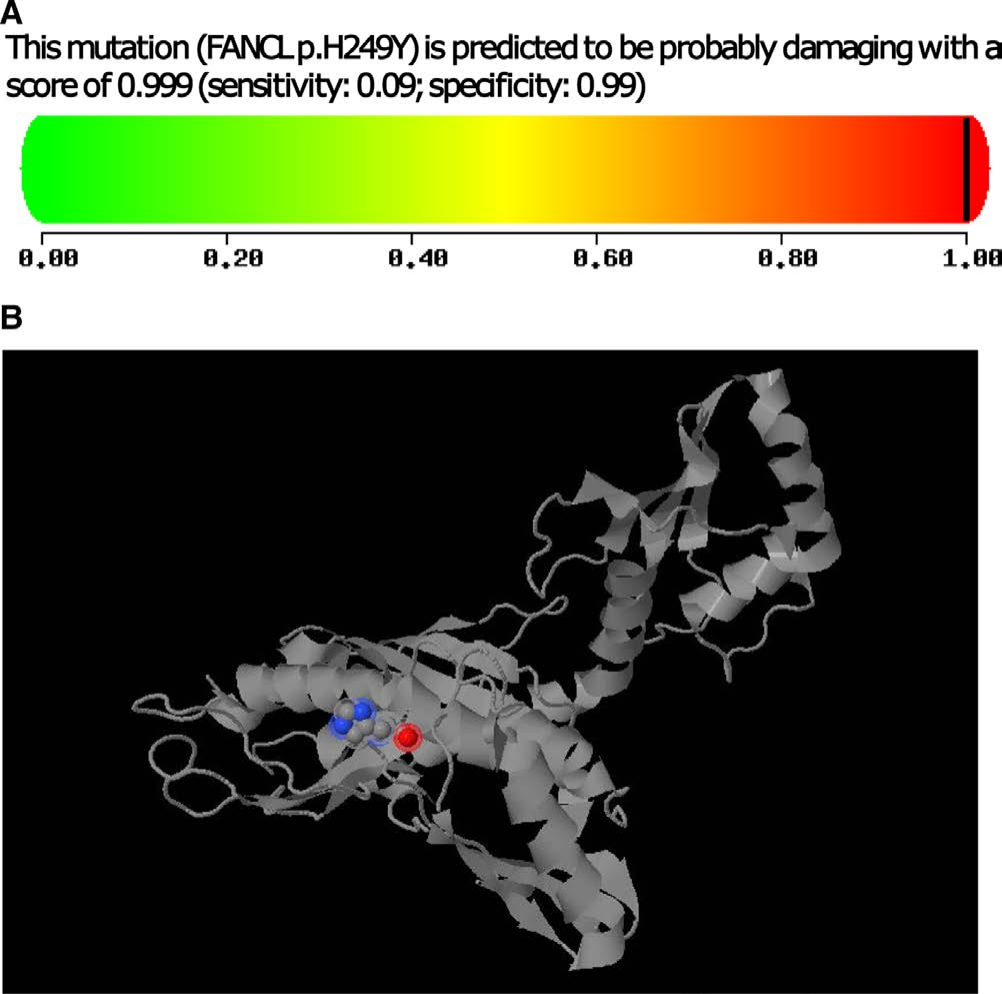
Function prediction diagram of p.H249Y mutation in FANCL. (A) The possibility of the FANCL protein function damage caused by p.H249Y mutation. The closer the value is to 1, the greater the possibility of damage. (B) 3D image of the damaged FANCL protein caused by p.H249Y mutation predicted by PolyPhen-2. The red part is the changed amino acid. FANCL = FA complementation group L.

FANCL is an E3 ubiquitin ligase and is a component of the multiprotein core complex in the FA pathway.^[12,13]^ FANCL interacts with FANCT, which serves as the E2-ubiquitin conjugating enzyme, and together they participate in the transfer of ubiquitin moieties to FANCD2 and FANCI. Ubiquitylation of FANCD2 and FANCI is necessary to activate the subsequent steps of the FA pathway which repair the DNA damage caused by interstrand crosslinks.^[11,13]^ The Functionally inactive mutation of FANCL gene (Exon9, c.745C > T, p.H249Y) we found in this case has never been reported. This mutation was located in the 9th exon of FANCL gene, and the amino acid at position 745 was mutated from C to T, resulting in the change of amino acid at position 249 from histidine to lysine. According to the prediction of PolyPhen-2 website (http://genetics.bwh.harvard.edu/pph2/), this mutation can lead to functional damage of the FANCL protein (Fig. 3). Currently, fewer FANCL mutation sites have been reported, and all of them are homozygous or compound heterozygous mutations in FA cases.^[14–20]^ We summarize these mutation sites in Table 1.

**Table 1:** FANCL mutation sites in FA that have been reported.

Serial number of mutation type	Mutation 1		Mutation 2		Heterozygosity or homozygous	Number of cases
	Base sequence	Amino acid sequence	Base sequence	Amino acid sequence		
1	c.1007_1009delTAT	p.Ile336_Cys337delinsSer	c.1095_1098dupAATT	p.Thr367AsnfsX13	Compound heterozygosity	1^[14]^
2	c.755_756insAT	p.M252fs	c.755_756insAT	p.M252fs	Homozygous	1^[15]^
3	c.375-2033C > G (intron)	–	c.1007_1009delTAT	p.I336-C337delinsS	Compound heterozygosity	1^[16]^
4	c.1092G > A	p.K364K	c.1092G > A	p.K364K	Homozygous	1^[16]^
5	c.375-2033C > G (intron)	–	c.871_874delGATT	p.D291Ffs*49	Compound heterozygosity	1^[16]^
6	c.268del	p.Leu90Phefs*6	c.268del	p.Leu90Phefs*6	Homozygous	2^[17]^
7	c.430del	p.Ser144Leufs*6	c.430del	p.Ser144Leufs*6	Homozygous	1^[17]^
8	c.822_823insCTTTCAGG	p.Asp275LeufsX13	c.822_823insCTTTCAGG	p.Asp275LeufsX13	Homozygous	1^[18]^
9	c.1092G > A	p.Trp341_Lys364del	c.1092G > A	p.Trp341_Lys364del	Homozygous	12^[19]^ + 2^[20]^
10	c.1092G > A	p.Trp341_Lys364del	c.592delA (intron)	–	Compound heterozygosity	1^[19]^

FA = Fanconi anemia, FANCL = FA complementation group L.

We reported for the first time an acquired BMF case with FANCL heterozygous mutation. We infer that heterozygous mutation of FANCL may play a role in the pathogenesis of this patient. The patient had a history of benzene exposure for half a year before the onset of the disease. Studies have reported that heterozygous mutations in FANC genes do not cause the onset of FA, but can lead to increased susceptibility to tumors and acquired BMF.^[8–11]^ We consider that the onset process of this patient is described below. Firstly, benzene induced genetic instability in the patient, followed by heterozygous mutation in FANCL leading to impaired function of DNA repair and the occurrence of acquired BMF. The patient’s father and brother also had this heterozygous mutation of FANCL gene, but they did not develop the disease, which may be related to their lack of exposure to factors that induce genetic instability. This case suggests that heterozygous mutations in FANCL gene may be associated with increased susceptibility to acquired BMF. However, further case collection and corresponding basic research are needed.

Based on current reports^[8–11]^ and this case report, we speculate that heterozygous mutations in the FANC gene may exist in a certain proportion of tumor and acquired BMF patients, but have not been detected. We recommend routine screening for FANC gene mutations in tumor and acquired BMF patients in clinical practice. If positive results are found, further screening can be conducted on their families.

## Acknowledgments

We gratefully acknowledge the financial support by the Natural Science Foundation of Sichuan Province (grant no. 2022NSFSC1330) and the Science and Technology Plan Project of Sichuan Province (grant no. 2019YJ0276).

## Author contributions

**Data curation:** Nan Zhang.

Formal analysis: Nan Zhang.

Funding acquisition: Nan Zhang, Xiao Wang.

Investigation: Nan Zhang, Xiao Wang, Xiao-Juan Miao, Xu-Pai Zhang, Xin-Yu Xia, Li Li.

Writing – original draft: Nan Zhang.

Writing – review & editing: Hao-Ping Sun.
